# Natural antioxidants from some fruits, seeds, foods, natural products, and associated health benefits: An update

**DOI:** 10.1002/fsn3.3217

**Published:** 2023-01-13

**Authors:** Md. Mizanur Rahaman, Rajib Hossain, Jesús Herrera‐Bravo, Mohammad Torequl Islam, Olubunmi Atolani, Oluyomi Stephen Adeyemi, Olubukola Abibat Owolodun, Learnmore Kambizi, Sevgi Durna Daştan, Daniela Calina, Javad Sharifi‐Rad

**Affiliations:** ^1^ Department of Pharmacy Bangabandhu Sheikh MujiburRahman Science and Technology University Dhaka Bangladesh; ^2^ Departamento de Ciencias Básicas, Facultad de Ciencias Universidad Santo Tomas Talca Chile; ^3^ Center of Molecular Biology and Pharmacogenetics, Scientific and Technological Bioresource Nucleus Universidad de La Frontera Temuco Chile; ^4^ Department of Chemistry University of Ilorin Ilorin Nigeria; ^5^ Department of Biochemistry, Medicinal Biochemistry, Infectious Diseases, Nanomedicine& Toxicology Laboratory Landmark University Omu‐Aran Nigeria; ^6^ Cell Biology and Genetics Unit, Department of Zoology University of Ilorin Ilorin Nigeria; ^7^ Department of Horticulture Cape Peninsula University of Technology Bellville South Africa; ^8^ Department of Biology, Faculty of Science Sivas Cumhuriyet University Sivas Turkey; ^9^ Beekeeping Development Application and Research Center Sivas Cumhuriyet University Sivas Turkey; ^10^ Department of Clinical Pharmacy University of Medicine and Pharmacy of Craiova Craiova Romania; ^11^ Facultad de Medicina Universidad del Azuay Cuenca Ecuador

**Keywords:** antioxidants, chronic diseases, free radicals, health benefits, inflammation, oxidative stress

## Abstract

Antioxidants are compounds that inhibit the oxidation of other molecules and protect the body from the effects of free radicals, produced either by normal cell metabolism or as an effect of pollution and exposure to other external factors and are responsible for premature aging and play a role in cardiovascular disease. degenerative diseases such as cataracts, Alzheimer's disease, and cancer. While many antioxidants are found in nature, others are obtained in synthetic form and reduce oxidative stress in organisms. This review highlights the pharmacological relevance of antioxidants in fruits, plants, and other natural sources and their beneficial effect on human health through the analysis and in‐depth discussion of studies that included phytochemistry and their pharmacological effects. The information obtained for this review was collected from several scientific databases (ScienceDirect, TRIP database, PubMed/Medline, Scopus, Web of Science), professional websites, and traditional medicine books. Current pharmacological studies and evidence have shown that the various natural antioxidants present in some fruits, seeds, foods, and natural products have different health‐promoting effects. Adopting functional foods with high antioxidant potential will improve the effective and affordable management of free radical diseases while avoiding the toxicities and unwanted side effects caused by conventional medication.

## Graphical abstract

Antioxidants are commonly used to be as supplements in food and also have been examined for inhibition of various diseases such as heart disease and cancer. Exogenous types of antioxidants such as vitamins, flavonoids, anthocyanins, and some mineral compounds are derived from natural sources but also obtained in synthetic forms, like butylhoxyanisole, butylhroxytoluene, and gallates, which are primarily synthetic. Antioxidants are getting prominence, particularly those established to prevent the alleged harmful impact of free radicals in the human body, and also the degradation of lipids and other nutritional elements. The antioxidant activities of some fruit and vegetables are herein discussed.

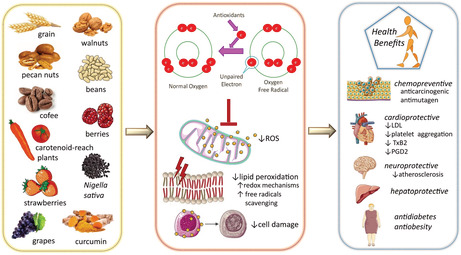

## 1 INTRODUCTION

Antioxidants are the most important substances that help to prevent the oxidation process. Oxidation is partly referred to as a chemical reaction capable of producing free radicles, as a result, chain reactions may occur, potentially causing serious damage to organisms' cells (Mititelu et al., 2020; Sharifi‐Rad, Kumar, et al., 2020). Antioxidants are compounds that scavenge free radicals in the human system. While the human body has a natural antioxidant defense system that keeps free radicles in check. Natural antioxidants found in food, particularly fruits, vegetables, and other plant‐based diets, plays important role in disease prevention (Popović‐Djordjević et al., 2022; Sharifi‐Rad, Dey, Koirala, et al., 2021). Antioxidants that are a word refer to two types of substances: industrial chemicals that are added to commodities to protect naturally occurring substances from oxidation substances found in foods and tissues (Quetglas‐Llabrés et al., 2022; Salehi et al., 2021). Industrial antioxidants, on the other hand, have a lot of applications, including oxidation inhibitors in fuels and preservatives in food and cosmetic products (Buga et al., 2019; Docea et al., 2020). Antioxidants are capable to end‐up chain reactions by removing the intermediates of free radicals. They perform the antioxidant characteristics by the way they are being oxidized, hence the antioxidants can be considered reducing agents. Some examples of this substance are thiols, ascorbic acid, and polyphenols (Sharifi‐Rad, Quispe, Imran, et al., 2021). Antioxidants are commonly used to be as supplements in food and also have been examined for inhibition of various diseases such as heart disease and cancer. Exogenous types of antioxidants such as vitamins, flavonoids, anthocyanins, and some mineral compounds are derived from natural sources but also obtained in synthetic forms, like butylhoxyanisole, butylhydroxytoluene, and gallates which are primarily synthetic. Antioxidants are getting prominence, particularly those established to prevent the alleged harmful impact of free radicals in the human body, and also the degradation of lipids and other nutritional elements (Sharifi‐Rad, Rodrigues, et al., 2020). The antioxidant activities of some fruit and vegetables are herein discussed.

## 2 METHODOLOGY

**FIGURE 1:**
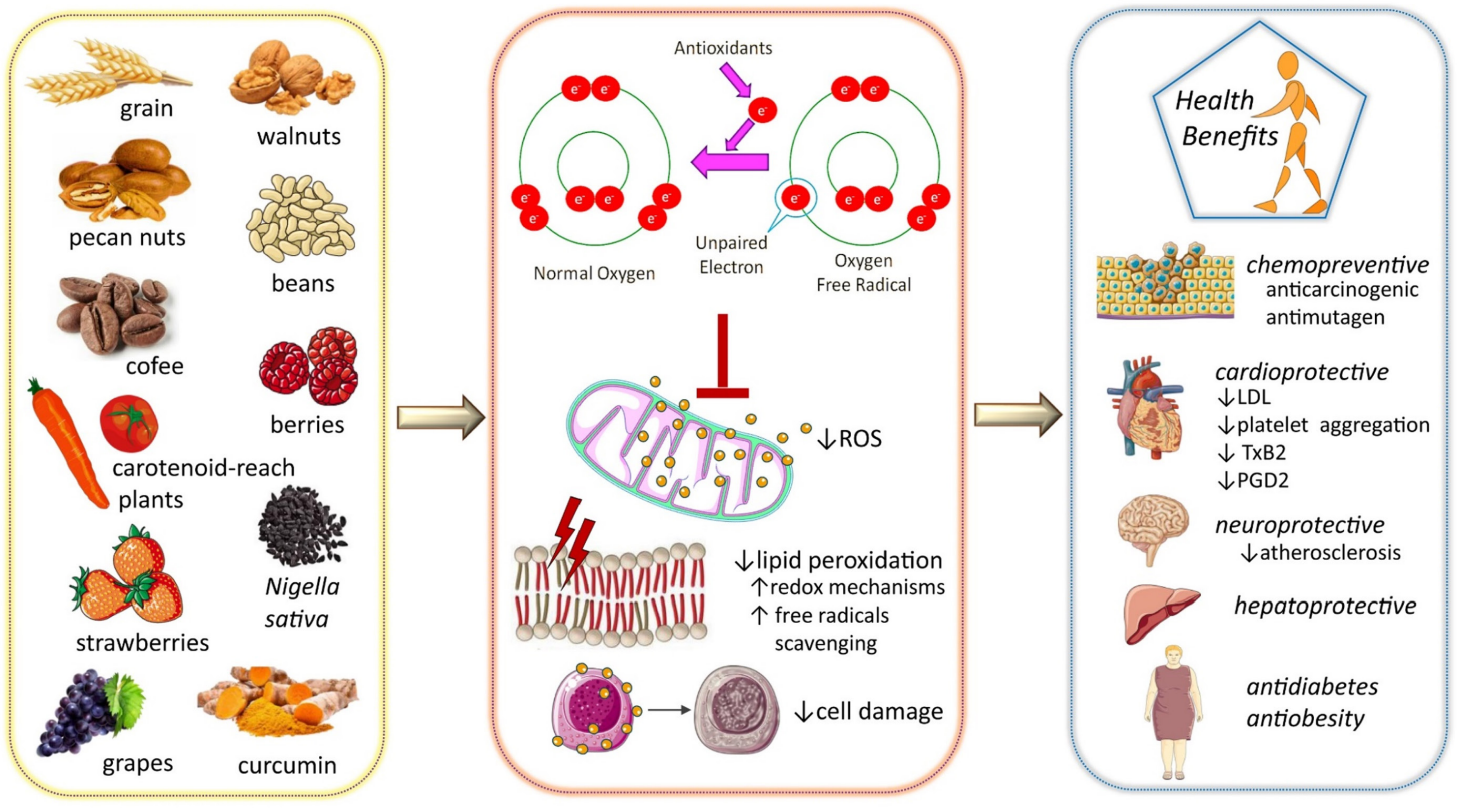
Antioxidant mechanisms of bioactive compounds from natural sources. Abbreviations and symbols: ↑ increase, ↓decrease, ROS reactive oxygen species, LDL low‐density lipoprotein, PGD2 prostaglandin D_2_, TxB2 thromboxane B2.

A detailed database search was conducted to identify recent articles that illustrate the effectiveness of antioxidants in preventing human disease. Data were searched in several online databases such as ScienceDirect, TRIP database, PubMed/Medline, Scopus, and Web of science using the following MeSH terms: Antioxidants/isolation & purification, Antioxidants/analysis, Biological Products/pharmacology, Antioxidants/pharmacology, Carotenoids/ isolation & purification, Carotenoids/pharmacology, carotenoids/analysis, food, Free Radical Scavengers/isolation & purification, Free Radical Scavengers/analysis, Oxidation–Reduction/drug effects, Free Radical Scavengers/pharmacology, Medicinal/chemistry, Plants, Polyphenols /pharmacology, Polyphenols/analysis, Polyphenols/isolation & purification. The names of the scientific species have been validated using the PlantList and chemical structures using ChemSpider (Heinrich et al., 2020; The Plant Lists, n.d). The most important antioxidant mechanisms have been summarized in Table 1 and Figure 1.

**TABLE 1:** Antioxidant potentials of fruits, plants, and natural compounds

Sources	Antioxidant compounds	Dose/conc. (R/A)	Potential mechanism of action	References
Apple	Phenolics, flavonoids	290.2 ± 4.2 mg–219.8 ± 1.8 mg (phenolics) 142.7 ± 3.7–97.6 ± 3.9 mg (flavonoids)	↓ Tumor cells growth(	Eberhardt et al. (2000)
Pecan nuts	Ellagic acid, galic acid, protocatechuic, p‐hydroxybenzoic acids	–	Pecan nut shell infusion has a high total phenolic compound and condensed tannins ↑ antioxidant activity is measured using various techniques	do Prado et al. (2009)
Coffee brews	Polyphenols, melanoidins	–	↑ Active oxygen‐scavenging activity	Cammerer and Kroh (2006)
Grape juice	Anthocyanins	25.56–460 mg/L	↓ Oxidative damage of cells	Burin et al. (2010), Munoz‐Espada et al. (2004)
Walnut (*Juglans regia* L.)	Phenolics	32.61 mg/g of GAE (cv. Mellanaise) to 74.08 mg/g of GAE t (cv. Franquette)	Vital in obtaining a visible supply of chemicals having antibacterial activity and health‐protective effects	Oliveira et al. (2008)
Berry	Anthocyanins	–	Health maintenance chemopreventive	Loliger (1991)
*Nigella sativa*	Thymoquinone, carvacrol, t‐anethole, 4‐terpineol	1.0 μg/ml	Effective ‐OH radical scavenging agents were used in the non‐enzymatic lipid peroxidation in liposomes and the deoxyribose degradation assay.	Burits and Bucar (2000)
Sesame coat (*Sesamum indicum* L.)	Sesamin sesamolin	–	Termination of free radical reactions ↑metal‐binding capabilities ↓ROS	Changa et al. (2002)
*Propolis* sp.	Kaemperol phenethyl caffeate	–	Prevents inflammation, heart disease, diabetes, and cancer	Kumazawa et al. (2004)
*Curcuma longa*	Curcumin I, Curcumin II, Curcumin III	20 μg/ml, 14 μg/ml, 11 μg/ml	↓ Lipid peroxidation	Ruby et al. (1995)
Ginger (*Zingiber officinale*)	Phenols	870.1 mg/g	↓ Lipid peroxidation	Stoilova et al. (2007)
Tomato	Lycopene, phenolics, flavonoids vitamins C, E	–	To get the most health advantages from tomatoes, eat them whole, including the skin and seeds	Al‐Wandawi et al. (1985)
Coriander (*Coriandrum sativum* L.)	Monoterpenoid, linalool	–	Inhibitory effect against radical‐scavenging characteristics that is the concentration‐dependent manner	Wangensteen et al. (2004)
Grain	Ferulic acid diferulic acids	–	Consumption of high‐fiber, whole‐grain diets has been linked to a lower risk of cancer and coronary heart disease	Adom and Liu (2002)
Carotenoid‐rich plants	β‐carotene	–	Physical quenching appears to play a substantial role in protecting biological systems from O^2−^mediated damage; the rate of the chemical process accounts for only 0.05% of the activity	Krasnovakii and Paramonava (1983)

## 3 ANTIOXIDANT ACTIVITY

### 3.1 Apples

According to a study, the formation of colon and liver cancer cells in vitro is inhibited by apple extracts in a dose‐dependent manner, and 100 g of well‐cleaned apples exhibits antioxidant capacity equivalence to 1500 mg of vitamin C (Eberhardt et al., 2000). The quantity of phenolics and flavonoids in Red Delicious apples extracted with 80% acetone was determined (Singleton & Rossi, 1965): the fresh apple extracts contained 290.2 ± 4.2 mg and 219.8 ± 1.8 mg phenolics compounds and also 142.7 ± 3.7 mg and 97.6 ± 3.9 mg flavonoids substance per 100 g of apples with and without peel (Eberhardt et al., 2000). Apple extracts with skin showed a significant reduction in tumor cell growth when compared with extracts without skin. The apple extracts showed no cytotoxicity at all of the doses examined (Eberhardt et al., 2000).

### 3.2 Grain

**FIGURE 2:**
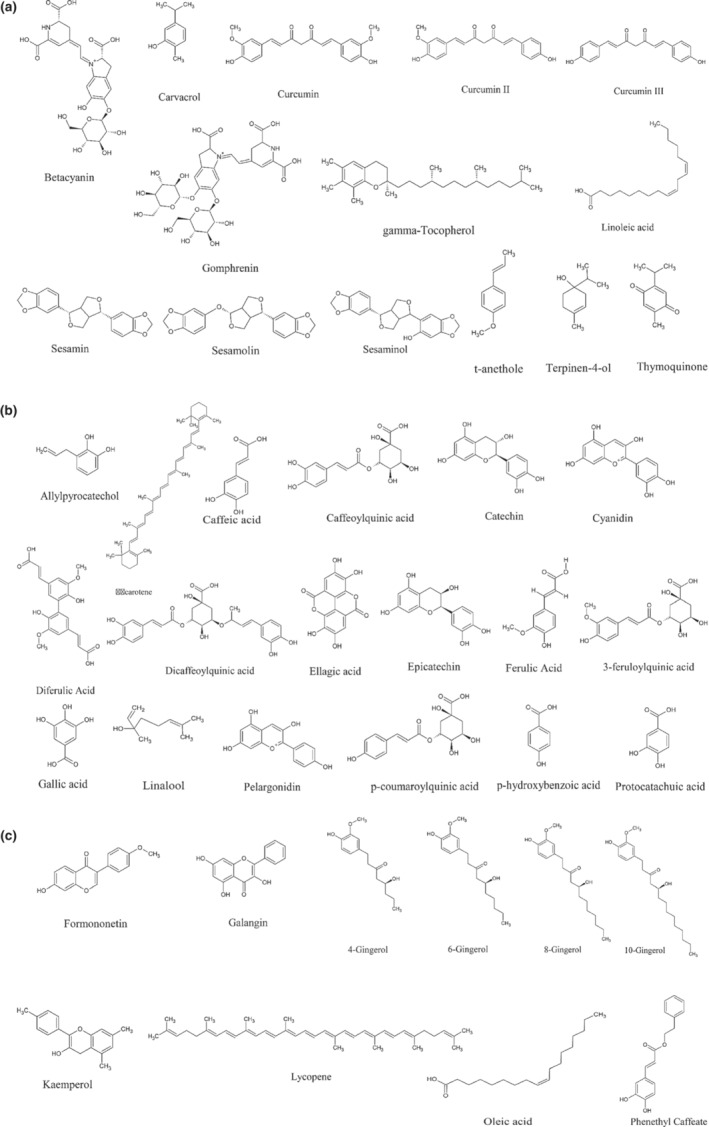
(a–c) The chemical structures of the most representative bioactive compounds with antioxidant effects.

Grains include several phytochemicals that benefit humans health through a variety of mechanisms, including antioxidants and hormone mediation (Sharifi‐Rad, Quispe, Imran, et al., 2021; Tsoukalas et al., 2019a). Whole grains have been demonstrated to lower the risk of developing colon cancer, breast cancer, diabetes, coronary heart disease, and overall mortality in various studies (Quispe et al., 2022) According to Thompson (1994), lignans and phytoestrogens found in grains may lower the incidence of different types of hormone‐related disorders like prostate cancer and breast cancer. Andreasen et al. (2001) suggest that both human and rat gastrointestinal esterase (that is usually found in intestinal mucosa and microbiota) may liberate ferulic and diferulic acids from cereal bran (Figure 2). These compounds have a highly potent antioxidant ability, and also these compounds' absorption level into the blood plasma has been established (Andreasen et al., 2001).

### 3.3 Carotenoid‐rich plants

Carotenoids are powerful antioxidants that protect the body from oxidative damage. Carotenoids are used as the most common natural pigments, and β‐carotene has been the most prominent compound with over 600 compounds so far (Olson & Krinsky, 1995) (Figure 2). Carotenoids found in the plant such as carrots, spinach, and tomatoes serve as an antioxidant in animals, and also as the provitamin. Carotenoids are a kind of vitamin A that can be found in plants. Several epidemiological studies have revealed that increasing carotenoids in one's diet reduces the risk of acquiring a range of degenerative diseases, such as cancer, cardiovascular disease, and ophthalmological disease (Mayne, 1996). Carotenoids affect cellular signaling and may activate redox‐sensitive regulatory mechanisms (Stahl & Sies, 2003). It is possible to gain a better understanding of carotenoids' potential involvement as prooxidants, as well as the significance of prooxidant activity in undesired reactions.

### 3.4 Pecan nut

The pecan nut *Carya illinoinensis* (Wangenh.) K. Koch is a member of the Juglandaceae family that grows in the place of southern United States and also in northern Mexico (Hanock, 1997). High amounts of monounsaturated and polyunsaturated fatty acids and also low levels of saturated fatty acids are rich in pecan nuts. It was also revealed that bioactive substances including sterols and tocopherols, and also a large amount of a total number of phenolic compounds, such as gallic acid, ellagic acid, protocatechuic, p‐hydroxybenzoic acids, and catechin with potential natural antioxidant activity, were present (de la Rosa et al., 2011; Kornsteiner et al., 2006). According to do Prado et al. (2009), pecan nuts contain a high fiber content (48% ± 0.06), with a total amount of phenolic content ranging from 116 to 167 mg GAE/g, and condensed tannin content ranging from 35 to 48 mg CE/g. In the ABTS system, antioxidant activity ranged from 1112 to 1763 mol TEAC/g. The antioxidant activity was reported to range from 305 to 488 mg TEAC/g (30 min reaction) and from 482 to 683 mg TEAC/g (24 h reaction) by using the DPPH technique. In the β‐carotene/linoleic acid system, the range of oxidation inhibition percentage is from 70% to 96%. Pecan nutshell infusion had a significant phenolic content and high antioxidant activity, according to the findings.

### 3.5 Coffee brews

Coffee beans and coffee beverages have long been recognized as effective antioxidants, and have been studied using various detection methods (Borelli et al., 2002; Daglia et al., 2000; Del‐Castillo et al., 2002; Steinhart et al., 2001). Recent research has proved that polyphenols in coffee play an important role in their potent antioxidant properties (Tsoukalas et al., 2021). Some of the known chemical compounds are present in coffee beans such as caffeoylquinic acids, feruloylquinic acids, dicaffeoylquinic acids, and p‐coumaroylquinic acids (Farah & Donangelo, 2006; Figure 2). Cammerer and Kroh (2006) show that stabilized radical EPR spectroscopy has used the determination of the overall antioxidant effect of coffee beverages. Depending on whether stabilized radical is used, the readings of Fremy's salt (potassium nitrosodisulfonate) or 2,2,6,6‐tetramethyl‐1‐piperidin‐1‐oxyl (TEMPO) may differ significantly. The radical marker TEMPO appears to be the superior radical marker for determining the antioxidant activity of Maillard reaction products in coffee. As a result, both major antioxidant active substances (polyphenols and melanoidins) particularly the ratio that vary depending on roasting conditions may play a significant role. Particular changes in antioxidant activity in coffee brews are shown to be time‐dependent during storage tests.

### 3.6 Chocolate

Chocolate is very popular in many western countries including the USA and Europe. It can only consume a small portion of total energy and fat. High sugar and caffeine content in chocolate provide a fast‐absorbing stimulant energy source and a sustainable energy source from high‐fat ingredients. Chocolate flavonoids and phenolics protect fat against rancidification, and also reduce the need for preservatives. The majority of flavonoids are strong antioxidants for LDL (low‐density lipoprotein) oxidation (Teissedre et al., 1996) and their administration is considered to be strongly related to coronary heart disease (Hertog et al., 1993). According to Waterhouse et al. (1996), cocoa powder extract is a powerful antioxidant that prevents LDL oxidation. Cocoa phenols reduced oxidation by 75% at 5 μmol/L GAE, whereas pure catechin (5 μmol/L) helps to inhibit oxidation by 87%.

### 3.7 Strawberries

Recent epidemiological studies have associated a diet rich in fruits and vegetables with a lower risk of cardiovascular disease and cancer (Salehi et al., 2020; Sharma et al., 2022). Strawberries (*Fragaria ananassa*) are widely consumed, both fresh and processed, and so provide a significant source of different compounds with significant health benefits against different diseases. For example, phenolic compounds with their antioxidative and antiproliferative properties (Meyers et al., 2003; Riboli & Norat, 2003). Because of reduced oxidation of low‐density lipoprotein and platelet aggregation, strawberry extracts and their components have been shown to have anti‐cancer, anti‐inflammatory, and heart disease prevention characteristics (Hannum, 2004; Sharifi‐Rad, Rodrigues, et al., 2020).

### 3.8 Grape juice

Phenolic compounds are abundant in grapes, especially red grapes (Fuleki & Ricaardo‐da‐Silva, 2003). Phenolic compounds are studied for their health benefits as well as their involvement in the production of grape products (Bub, 2003; O'Byrne, 2002). They have different types of antioxidant properties and also the ability to protect cells from oxidative damage (Cavallini et al., 1978). In multiple epidemiological and clinical studies, eating fruits and fresh vegetables high in polyphenols has been found to decrease the risk of cardiovascular disease and cancer (Garcia‐Alonso, 2004). According to Burin et al. (2010), the antioxidant activity of all juices tested using the DPPH technique ranged from 2.51 to 11.05 mM. In other research, the antioxidant efficiency of red wine in 16 samples was evaluated using the DPPH technique, with results ranging from 6.10 to 17.41 mM (Li et al., 2006).

### 3.9 *Juglans regia* L.

Walnut (*Juglans regia* L.) is a valuable nut crop that is widely consumed across the world. Several studies have proven the antioxidant properties of walnut products, particularly fruits (Li et al., 2007), leaves (Pereira et al., 2007), and liqueurs that are produced from green fruits (Pereira et al., 2008). Green walnuts, shells, bark, green walnut husks (epicarp), kernels, and leaves, in addition to dry fruit (nuts), have demonstrated positive benefits in the cosmetic and medicinal industries (Stampar et al., 2006). Walnut husk extracts in their green form have significant reducing power. The capacity of a substance to reduce may be a good indication of its potent antioxidant effect (Meir et al., 1995). Oliveria et al. (2008) used three different types of assays to determine the antioxidant ability of walnut green husk samples against ROS species: scavenging activity on DPPH radicals, reducing power, and lipid peroxidation inhibition via the β‐carotene‐linoleate system. The antioxidant capacity of aqueous extracts of green walnut was investigated using a reducing power test, scavenging effects on DPPH (2, 2‐diphenyl‐1‐picrylhydrazyl) radicals, and a β‐carotene linoleate model system. In reducing power and DPPH assays, all of the examined extracts had EC50 values less than 1 mg/ml, indicating a concentration‐dependent antioxidative capacity.

### 3.10 Aronia melanocarpa

Berries are a type of plant material that is high in phenolics. Berries and fruits are high in flavonoids and phenolic acids, which have antioxidant properties. The antioxidant capacity of plasma was considerably improved by eating a controlled diet high in fruits and berries (Cao et al., 1998). According to several epidemiological research, there is a strong negative relationship between fruit and vegetable intake and mortality due to heart diseases (Hertog et al., 1993; Knekt et al., 1996). As the future trend moves toward fruits with specific health effects, scientists, food manufacturers, and consumers are becoming more interested in the antioxidant contents of berries, which maintain health and protect us from coronary heart disease and cancer (Loliger, 1991). *Aronia melanocarpa* berries contain a lot of o‐phenolics such as caffeic acids, (−) epicatechin, cyanidin, and quercetin derivatives. The presence of an o‐dihydroxy structure in the B ring confers increased radical stability and participates in electron delocalization, these compounds are the most active antioxidants (Rice‐Evans et al., 1995).

### 3.11 *Phaseolus vulgaris* L.

Dry beans, commonly known as *Phaseolus vulgaris*, can lower the risk of diabetes and obesity (Geil & Anderson, 1994) due to their significant different activity on the blood sugar and insulin response, and therefore their potential utility for diabetes prevention and control (Sandberg, 2000). Dry beans have also been found to have significant activity against coronary heart disease (Anderson et al., 1984; Bazzano et al., 2001). Hughes et al. (1997) provide evidence that supports prior epidemiological research that has linked high levels of dry bean consumption to reduce the risk of colon cancer. Discovered that while having lower antioxidant capacity compared with other types of bean fractions obtained by the way of dry dehulling, bean hulls exhibited considerable antioxidant capacities, as determined by their ability to scavenge free radical or inhibit the lipid peroxidation process. The antioxidant properties of manually separated hulls and their fractional methanolic extracts may account for some of the antimutagenic properties observed.

### 3.12 *Nigella sativa* L.

*Nigella sativa* L. is an annual Ranunculaceae herbaceous plant whose seeds have been traditionally used for the treatment of asthma, bronchitis, cough, rheumatism, headache, fever, eczema, influenza, and as diuretic, lactagogue, and vermifuge in Middle East, Northern Africa, and India; we understood very little about the volatile oil activity of *N. sativa* (Mahmoud & Shaheen, 1996). Preliminary investigations (Houghton et al., 1995) revealed that the essential oil's primary constituent, thymoquinone, inhibits the non‐enzymatic lipid peroxidation activity in liposomes. Both non‐enzymatic lipid peroxidation assays and the deoxyribose test demonstrated donor features in the DPPH assay and hydroxyl radical scavenging qualities (Aruoma & Cuppett, 1997). In the experiments conducted on‐site, neither essential oil nor the substances carvacrol, thymoquinone, t‐anethole, or 4‐terpineol, which all are contributing to the volatile fraction's radical scavenging function in various ways, showed pro‐oxidant activity (Aruoma, 1991; Gutteridge et al., 1981; Halliwell, 1993). Burits and Bucars' (2000) study showed that *N. sativa* has radical scavenging action, implying that using black cumin seeds to treat various inflammatory illnesses could be beneficial and reasonable.

### 3.13 Beta vulgaris

Red beet is one of the major sources of chemical constituent betalains, which are mostly used in the current food industry. The betalains are essential natural colorants that were among the first to be discovered to be used in food production (Francis et al., 1999). Much research on the antioxidative and antiradical action of betalains (mostly betanin) from beetroots (*Beta Vulgaris*) has recently been reported (Escribano et al., 1998). Because of the health benefits of red beet goods, including them in one's diet regularly may protect against some oxidative stress‐related illnesses in humans (Kanner et al., 2001). Cai et al.'s (2003) study provides evidence that betalains from of the Amaranthaceae family of plants, primarily red‐violet gomphrena category betacyanins and yellow betaxanthins, have extremely high antioxidant activity when compared with the traditional antioxidants (ascorbic acid, catechin, and rutin), indicating that betalains could be a good source including both antioxidants as well as natural colorants.

### 3.14 *Sesamum indicum* L.

Sesame (*Sesamum indicum* L.) is a significant oilseed crop grown border between India, Sudan, China, and Myanmar, accounting for 60% of global sesame yield (Abou‐Gharbia et al., 1997). Budowski (1964) said that sesame oil is highly prone to degradation in comparison with other types of vegetable oils. Sesame oil is stable due to the presence of important chemical constituents such as sesamin, sesaminol, sesamolin, sesame, and g‐tocopherol (Shahidi et al., 1997). Changa et al.'s (2002) study provides evidence that sesame coat exhibits anti‐oxidant activity against a broad of lipid peroxidation process in vitro. The numerous antioxidant processes of sesame coats can be due to their strong hydrogen‐donating capacity, metal‐chelating ability, and efficacy as hydroxyl radical scavengers.

### 3.15 *Piper betle* L.

*Piper betle* L. (Piperaceae) is a plant that is widely used as a masticatory agent in Asia and its leaves have also a strong pungent aromatic flavor. The leaves show some therapeutic properties such as digestive and stimulants. Medicinally the leaves *Piper betle* L. is necessary for catarrhal and pulmonary affections (The Wealth of India, 1969). The phenolic component allylpyrocatechol found in the leaves has been showing significant activity against halitosis‐causing obligate oral anaerobic organisms (Ramji et al., 2002). The leaves of *P. betle* also had an efficient hepatoprotective effect and increased tissue antioxidant status by enhancing non‐enzymatic antioxidant activity (reduced glutathione, vitamin E and vitamin C) and also free radical‐detoxifying enzyme activities in ethanol‐treated rats' liver and kidney (Saravanan et al., 2002). Platelet aggregation was decreased by *P. betle* leaf extract, which had antioxidative effects as well as impacts on the formation of thromboxane B2 (TxB2) and prostaglandin‐D2 (PGD2) production (Jeng et al., 2002).

### 3.16 Caffeic acid‐rich plants

Caffeic acid (3, 4‐hydroxycinnamic acid) has been found to protect low‐density lipoprotein from α‐tocopherol damage (LDL) (Laranjinha et al., 1995). In a variety of systems, chlorogenic and caffeic acid conjugates are very powerful antioxidants (Fukumoto & Mazza, 2000; Meyer et al., 1998) (Figure 2). Caffeic acid and its many derivatives are known to be good polyphenol oxidase substrates, and they can oxidize plant tissues or plant‐derived products under certain conditions (Bassil et al., 2005). Gulcin's (2006) study provides evidence that in vitro studies such as total antioxidant activity by ferric thiocyanate technique, reducing power, ABTS• + scavenging, DPPH• scavenging, superoxide anion radical scavenging, and metal chelating activity give evidence that caffeic acid was the most potent antioxidant when compared with typical antioxidant chemicals like BHA, BHT, a natural antioxidant, α‐tocopherol and trolox, a water‐soluble homolog of tocopherol.

### 3.17 *Ocimum* sp.

Many Lamiaceae herb spices, including sage, oregano, and thyme, have high antioxidant properties (Hirasa & Takemasa, 1998). It includes 50 to 150 species of plants and shrubs in the Ocimum genus (Simon et al., 1999). Several phenolic compounds were found in the plant extracts of *Ocimum* sp. with their strong antioxidant activity (Nakatani, 1997). Javanmardi et al.'s (2003) study provided evidence that *Iranian Ocimum*, which is commonly found in Iranian foods, is a potent radical scavenger and can be used as a source of naturally found antioxidants in side dishes, medicine, and commercial types.

### 3.18 *Ceratonia siliqua* L.

For many years, the plant namely a carob tree (*Ceratonia siliqua* L.) has been planted widely in Mediterranean nations as the plant has high polyphenol content, particularly concentrated from tannins (Avallone et al., 1997). Due to their low cost and lack of caffeine, carob pods have mostly been utilized as a replacement for cocoa in few nations (Yousif & Alghzawi, 2000). At the same doses, the crude polyphenol fraction (CPP) had lower antioxidant activity than real polyphenol components in the DPPH free radical scavenging, erythrocyte ghost, and microsomal systems (Kumazawa et al., 2002). Kumazawa et al. (2002) provide evidence that the antioxidant activity of a crude polyphenol produced from carob pods has been demonstrated. CPP, in particular, appears to have a substantial anti‐discoloration impact on β‐carotene. Given that most carob pods are now discarded and ineffectively used, our findings imply that carob pods might be used as a functional food or food additive.

### 3.19 *Propolis* sp.

In many places around the world, *Propolis* sp. has been utilized in traditional medicine (Ghisalberti, 1979). Propolis is commonly used within food and beverage to promote good health and prevent diseases including inflammation, diabetes, heart disease, cancer etc. (Banskota et al., 2001). The Kumazawa et al.'s (2004) study shows that the antioxidant actions of *Propolis* sp. from different geographical sources, such as Argentina, Brazil, Australia, Bulgaria, China, Hungary, New Zealand, South Africa, Ukraine, Thailand, Uruguay, Chile, the United States, and Uzbekistan, differ. The antioxidant properties of ethanol extracts of propolis (EEP) were determined using the β‐carotene bleaching and 1.1‐diphenyl‐2‐picrylhydrazyl (DPPH) free radicle scavenging tests. The majority of the antioxidant components in EEP were discovered and quantified using the HPLC analysis with the effective photo‐diode array (PDA) and mass spectrometric (MS) detection. Antioxidant substances like kaempferol and phenethyl caffeate were found in *Propolis* sp. with high antioxidant activity.

### 3.20 Curcuma longa

Curcumin which is isolated from the plant *Curcuma longa* and has several pharmacological properties is mostly known to be a natural antioxidant (Quispe et al., 2022; Salehi et al., 2020). Curcumin has been found to have antimutagenic and anticarcinogenic properties due to its antioxidant potential. The National Cancer Institute is testing it as a chemopreventive agent (Quispe et al., 2022; Salehi et al., 2019). Curcumin is mostly composed of curcumin I (diferuloylmethane), but it also contains curcumin II (6%) and curcumin III (3%) (0.3%). Demethylated derivatives found from curcumin are one of the most powerful regulators of lipid peroxidation, while total methylation of these compounds results in the loss of antioxidant activity (Sharma, 1976). Curcumin I, curcumin II, and curcumin III can inhibit lipid peroxidation by 50% at concentrations of 20 pg/ml, 14 pg/ml, and 11 pg/ml, respectively. The synthetic type of derivatives of curcumin I (14 pg/ml) and curcumin III (13 pg/ml) was just as effective as the natural compounds (Ruby et al., 1995). Ruby et al.'s (1995) study provides evidence that the demethylation of curcumin, as seen in curcumin III, increases one's antioxidant activity, according to this study. Salicyl curcuminoid, a curcumin III isomer, has also yielded consistent results.

### 3.21 *Zingiber officinale* (L.) Rosc

Ginger (*Zingiber officinale* (L.) Rosc) is a flowering plant that has been widely used as a spice for over 2000 years (Bartley & Jacobs, 2000). The roots contain polyphenols compounds (especially 6‐gingerol and its derivatives), which have a potent antioxidant activity (Chen et al., 1986). In vitro analysis showed that ginger extract could ameliorate the effects of free radicals as well as the peroxidation of lipids. As a result, it may be able to prevent or reduce harm in a human system by acting as a free radical scavenger (Aruoma et al., 1997; Valko et al., 2004). Ginger extract consumption is expected to decrease the course of atherosclerosis because it is connected to reducing the macrophage‐mediated oxidation of LDL, decreasing absorption of oxidized LDL by macrophages, reducing the oxidative status of LDL, and finally reducing LDL aggregation (Fuhrman et al., 2000). Stoilova et al.'s (2007) study provided evidence that the ginger carbon dioxide extract contains a lot of polyphenols. It had a high DPPH‐scavenging potential as well as a significant lowering capability. The extract is thought to be useful as an antioxidant during the early stages of fat oxidation. At both lower and high temperatures at 37 and 80°C, the antioxidant activity of the ginger extract was equivalent to that of BHT in preventing lipid peroxidation. The stage of synthesis of secondary products of fat auto‐oxidation was perhaps the most hindered.

### 3.22 Solanum lycopersicum

Tomatoes (*Solanum lycopersicum*), sometimes known as Tomatoes, are versatile vegetables that can be eaten raw or cooked. Since various epidemiological studies have shown that regular consumption of fruits and vegetables, particularly tomatoes, may work to minimize cancer and cardiovascular disease, there has been an intense emphasis on the antioxidant compounds of tomatoes (Giovannucci, 1999; Heber, 2000; Rao & Agarwal, 2000). The antioxidant properties shown by raw tomatoes and processed tomato products are due to the presence of different essential components such as phenolics, lycopene, flavonoids, and vitamins C and E (Stwart et al., 2000). Al‐Wandawi et al. (1985) reported that in comparison with the tomato pulp and seeds, tomato peel has a high concentration of lycopene. Tomato skin and seeds were also found to contain necessary amino acids, with the tomato seeds containing particularly high levels of minerals (Fe, Mn, Zn, and Cu), as well as monounsaturated fatty acids (especially, oleic acid). Toor and Savage (1992) study provide evidence that antioxidant substances are abundant in the skin and seed extract. Incorporating skin and seed extracts into foods for home consumption or processed goods might result in an increase of 40%–53% in the level of all main antioxidants in the finished products.

### 3.23 Laminaria japonica

A sulfated polysaccharide found in seaweed has a different type of biological activities such as anticoagulant, anti‐inflammatory, antithrombotic, contraceptive, anticancer, antiviral, and antioxidant effects (Jhamandas et al., 2005; Patankar et al., 1993; Ponce et al., 2003; Tehila et al., 2005; Zhuang et al., 1995). Sulfated polysaccharide fractions have been reported to exhibit good antioxidant activities (Wang et al., 2008). Wang et al. (2008) further corroborated that the antioxidant properties were found in fucoidan and certain fractions isolated from *L. japonica*. The stated antioxidant ability, on the other hand, varies. In vitro, the majority of the fractions were more powerful antioxidants than fucoidan. However, a link was found between sulfate content and the ability to scavenge superoxide radicals.

### 3.24 *Coriandrum sativum* L.

Coriander (*Coriandrum sativum* L.; Umbelliferae) is a widely grown herb that is primarily grown for its seeds. The monoterpenoid linalool is the most important component of the essential oil found in the seeds (up to 1%) (Wichtl, 1994). The seeds are primarily responsible for coriander's medical usage, and it has been used to treat stomach, worms, rheumatism, and joint discomfort (Wichtl, 1994). Wangensteen et al.'s (2004) study provides evidence that Coriander seeds and leaves exhibit an inhibitory effect against radical‐scavenging characteristics in a concentration‐dependent manner. However, the impacts were more pronounced in leaf extracts than those in seeds. Incorporating both the seeds and leaves of coriander into the diet might boost antioxidant levels, preventing food from oxidizing.

## 4 THERAPEUTIC PERSPECTIVES AND LIMITATIONS

### 4.1 Health benefits of antioxidants

In the last decade, antioxidants have got a lot of hype for their role in reducing free radicals and oxidative stress, as well as cancer prevention and treatment (Sharifi‐Rad, Quispe, Durazzo, et al., 2022; Taheri et al., 2022; Tsoukalas et al., 2019b). In such circumstances, phenols and polyphenols are frequently of great interest; they can be detected using enzymes including tyrosinase or any other phenol oxidases, or perhaps even plant tissue carrying such enzymes (Hossain et al., 2021; Sharifi‐Rad, Quispe, Herrera‐Bravo, et al., 2021). A few common types of disease such as cancer, obesity, coronary heart disease, type 2 diabetes, cataract, and hypertension are induced by oxidative stress, and fruits, vegetables and so much less processed staple foods provide the best protection against these diseases (Konovalov et al., 2022; Painuli et al., 2022). The natural antioxidants included in fruits and vegetables have a favorable health effect, according to the explanation (Hossain et al., 2021; Painuli et al., 2022). Only a few of the antioxidants found in dietary plants include carotenoids, coumarins, phenolic compounds, stilbenes, benzoic acid derivatives, flavonoids, proanthocyanidins, and lignans (Islam et al., 2021). Blackberries, strawberries, walnuts, cranberries, artichokes, raspberries, brewed coffee, blueberries, pecans, ground cloves, grape juice, and unsweetened chocolate ranked at the top of the classification due to normal serving quantities (Kim et al., 2002). Antioxidants such as polyphenols, vitamin C, vitamin E, beta‐carotene, and lycopene are abundant in fruit juice and beverages (Islam et al., 2021; Varela et al., 2022). Fruit juice, beverages, and hot beverages have been shown to lower morbidity and mortality associated with degenerative diseases (Calina et al., 2020; Sharifi‐Rad, et al., 2022a).

### 4.2 Limitations

The term antioxidant refers to a chemical property of an electron‐donating substance (Padureanu et al., 2019; Sharifi‐Rad et al., 2022b). Antioxidants come in a variety of forms, each with a unique function in the body and action mechanism (Salehi et al., 2019; Sharifi‐Rad, Rodrigues, et al., 2020; Taheri et al., 2022). One common fallacy is that one antioxidant can indeed be substituted for another and have the same effect; nevertheless, each antioxidant has its biological qualities (Kasote et al., 2015; Scheau et al., 2021). There is also a clear differentiation between eating antioxidants and taking an isolated ingredient as a food supplement (Sharifi‐Rad et al., 2022b, 2022c). Single antioxidant quantities in food do not affect the total antioxidant capacity of the food (Benzie & Choi, 2014). The total antioxidant potential of food is mostly determined by synergic and redox interactions among the many types of molecules present (Butnariu et al., 2022; Semwal et al., 2022). The antioxidant performance of any fruit or vegetable is often dependent on the geographical location where they are grown. Many substances that give beneficial activity in laboratory pharmacological experiments do not work the same way when they are introduced into the human body (Akhtar, 2015). Furthermore, many natural antioxidants have low bioavailability. Antioxidants like polyphenols are sometimes found in such low concentrations in the blood that no discernible effect is seen (Alshehri et al., 2022; Sharifi‐Rad, Quispe, Bouyahya, et al., 2022). As a result, new approaches to increasing bioavailability such as incorporation into pharmaceutical nano‐formulations or changes in chemical structure are needed in the future (Patra et al., 2018).

## 5 CONCLUSION

The increasing interest in antioxidants from a natural source is apparently due to the associated health benefits. This exogenous source of potent antioxidants provides a readily available and affordable alternative for the management of oxidative stress‐related diseases induced by the attack of free radicles on key biological compounds such as lipids or nucleic acids. The safety profile of many natural sources of antioxidants, and the affordability, and availability of natural antioxidant sources make them a sustainable alternative for the present and the future.

## CONFLICT OF INTEREST

The authors declare that the research was conducted in the absence of any commercial or financial relationships that could be construed as a potential conflict of interest.

## Data Availability

Not applicable.

## References

[fsn33217-bib-0001] Abou‐Gharbia, H. A. , Shahidi, F. , Shahata, A. A. Y. , & Youssef, M. M. (1997). Effects of processing on oxidative stability of sesame oil extracted from intact and dehulled seeds. Journal of the American Oil Chemists' Society, 74, 215–221.

[fsn33217-bib-0002] Adom, K. K. , & Liu, R. H. (2002). Antioxidant activity of grains. Journal of Agricultural and Food Chemistry, 50, 6182–6187. 12358499 10.1021/jf0205099

[fsn33217-bib-0003] Akhtar, A. (2015). The flaws and human harms of animal experimentation. Cambridge Quarterly of Healthcare Ethics: CQ, 24, 407–419. 26364776 10.1017/S0963180115000079 PMC4594046

[fsn33217-bib-0201] Alshehri, M. M. , Quispe, C. , Herrera‐Bravo, J. , Sharifi‐Rad, J. , Tutuncu, S. , Aydar, E. F. , Topkaya, C. , Mertdinc, Z. , Ozcelik, B. , Aital, M. , Kumar, N. V. A. , Lapava, N. , Rajkovic, J. , Ertani, A. , Nicola, S. , Semwal, P. , Painuli, S. , González‐Contreras, C. , Martorell, M. , … Cho, W. C. (2022). A Review of Recent Studies on the Antioxidant and Anti‐Infectious Properties of Senna Plants. Oxidative Medicine and Cellular Longevity, 2022, 6025900. 10.1155/2022/6025900 35154569 PMC8837466

[fsn33217-bib-0004] Al‐Wandawi, H. , Abdul‐Rahman, M. , & Al‐Shaikhly, K. (1985). Tomato processing wastes as essential raw material sources. Journal of Agricultural and Food Chemistry, 33, 804–807.

[fsn33217-bib-0005] Anderson, J. W. , Story, L. , Sieling, B. , Chen, W. J. L. , Petro, M. S. , & Story, J. (1984). Hypocholesterolemic effects of oat‐bran or bean intake for hypercholesterolemic men. The American Journal of Clinical Nutrition, 48, 749–753. 10.1093/ajcn/40.6.1146 6095635

[fsn33217-bib-0006] Andreasen, M. F. , Kroon, P. A. , Williamson, G. , & Garcia‐Conesa, M. T. (2001). Intestinal release and uptake of phenolic antioxidant diferulic acids. Free Radical Biology & Medicine, 31, 304–314. 11461767 10.1016/s0891-5849(01)00585-8

[fsn33217-bib-0008] Aruoma, O. I. (1991). Free radicals and food additives (pp. 18–19). Taylor & Francis.

[fsn33217-bib-0009] Aruoma, O. I. , & Cuppett, S. L. (1997). Antioxidant methodology: In vivo and In vitro concepts (p. 10). AOCS Press.

[fsn33217-bib-0010] Aruoma, O. I. , Spencer, J. P. , Warren, D. , Jenner, P. , Butler, J. , & Halliwell, B. (1997). Characterization of food antioxidants, illustrated using commercial garlic and ginger preparations. Food Chemistry, 60(2), 49–156.

[fsn33217-bib-0011] Avallone, R. , Plessi, M. , Baraldi, M. , & Monzani, A. (1997). Determination of chemical composition of carob (*Ceratonia siliqua*): Protein, fat, carbohydrates, and tannins. Journal of Food Composition and Analysis, 10, 166–172.

[fsn33217-bib-0012] Banskota, A. H. , Tezuka, Y. , Adnyana, I. K. , Ishii, E. , Midorikawa, K. , Matsushige, K. , & Kadota, S. (2001). Hepatoprotective and anti‐*Helicobacter pylori* activities of constituents from Brazilian propolis. Phytomedicine, 8, 16–23. 11292234 10.1078/0944-7113-00004

[fsn33217-bib-0013] Bartley, J. , & Jacobs, A. (2000). Effects of drying on flavour compounds in Australian‐grown ginger (*Zingiber officinale*). Journal of the Science of Food and Agriculture, 80, 209–215.

[fsn33217-bib-0014] Bassil, D. , Makris, D. P. , & Kefalas, P. (2005). Oxidation of caffeic acid in the presence of l‐cysteine: Isolation of 2‐S‐cysteinylcaffeic acid and evaluation of its antioxidant properties. Food Research International, 38, 395–402.

[fsn33217-bib-0015] Bazzano, L. , He, J. , Ogden, L. G. , Loria, C. , Vupputuri, S. , Myers, L. , & Whelton, P. K. (2001). Legume consumption and risk of coronary heart disease in US men and women. Archives of Internal Medicine, 161, 2573–2578. 11718588 10.1001/archinte.161.21.2573

[fsn33217-bib-0016] Benzie, I. F. , & Choi, S. W. (2014). Antioxidants in food: Content, measurement, significance, action, cautions, caveats, and research needs. Advances in Food and Nutrition Research, 71, 1–53. 24484938 10.1016/B978-0-12-800270-4.00001-8

[fsn33217-bib-0017] Borelli, C. R. , Visconti, A. , Mennella, C. , Anese, M. , & Fogliano, V. (2002). Chemical characterization and antioxidant properties of coffee melanoidins. Journal of Agricultural and Food Chemistry, 50, 6527–6533. 12381145 10.1021/jf025686o

[fsn33217-bib-0018] Bub, A. (2003). Fruit juice consumption modulates antioxidative status, immune status, and DNA damage. Journal of Nutrition Biochemistry, 14, 90–98. 10.1016/s0955-2863(02)00255-3 12667600

[fsn33217-bib-0019] Budowski, P. (1964). Recent research on sesamin, sesamolin, and related compounds. Journal of the American Oil Chemists' Society, 41, 280–285.

[fsn33217-bib-0020] Buga, A. M. , Docea, A. O. , Albu, C. , Malin, R. D. , Branisteanu, D. E. , Ianosi, G. , Ianosi, S. L. , Iordache, A. , & Calina, D. (2019). Molecular and cellular stratagem of brain metastases associated with melanoma. Oncology Letters, 17, 4170–4175. 30944612 10.3892/ol.2019.9933 PMC6444343

[fsn33217-bib-0021] Burin, V. M. , Falcao, L. D. , Gonzaga, L. V. , Fett, R. , Rosier, J. P. , & Bordignon‐Luiz, M. T. (2010). Colour, phenolic content and antioxidant activity of grape juice. Food Science and Technology, 30, 1027–1032.

[fsn33217-bib-0022] Burits, M. , & Bucar, F. (2000). Antioxidant activity of *Nigella sativa* essential oil. Phytotherapy Research, 14, 323–328. 10925395 10.1002/1099-1573(200008)14:5<323::aid-ptr621>3.0.co;2-q

[fsn33217-bib-0204] Butnariu, M. , Quispe, C. , Herrera‐Bravo, J. , Sharifi‐Rad, J. , Singh, L. , Aborehab, N. M. , Bouyahya, A. , Venditti, A. , Sen, S. , Acharya, K. , Bashiry, M. , Ezzat S. M. , Setzer, W. N. , Martorell, M. , Mileski, K. S. , Bagiu, I. C. , Docea, A. O. , Calina, D. , Cho, W. C . (2022). The Pharmacological Activities of *Crocus sativus* L.: A Review Based on the Mechanisms and Therapeutic Opportunities of its Phytoconstituents. Oxidative Medicine and Cellular Longevity. 2022, 8214821. 10.1155/2022/8214821 35198096 PMC8860555

[fsn33217-bib-0023] Cai, Y. , Sun, M. , & Corke, H. (2003). Antioxidant activity of betalains from plants of the amaranthaceae. Journal of Agricultural and Food Chemistry, 51, 2288–2294. 12670172 10.1021/jf030045u

[fsn33217-bib-0205] Calina, D. , Buga, A. M. , Mitroi, M. , Buha, A. , Caruntu, C. , Scheau, C. , Bouyahya, A. , El Omari, N. , El Menyiy, N. , & Docea, A. O. (2020). The Treatment of Cognitive, Behavioural and Motor Impairments from Brain Injury and Neurodegenerative Diseases through Cannabinoid System Modulation‐Evidence from In Vivo Studies. Journal of Clinical Medicine, 9(8), 2395. 10.3390/jcm9082395 32726998 PMC7464236

[fsn33217-bib-0024] Cammerer, B. , & Kroh, L. W. (2006). Antioxidant activity of coffee brew. European Food Research and Technology, 223, 469–474.

[fsn33217-bib-0025] Cao, G. , Booth, S. L. , Ssdowski, J. A. , & Prior, R. L. (1998). Increases in human plasma antioxidant capacity after consumption of controlled diets high in fruit and vegetables. The American Journal of Clinical Nutrition, 68, 1081–1087. 9808226 10.1093/ajcn/68.5.1081

[fsn33217-bib-0027] Cavallini, L. , Bindoli, A. , & Siliprandi, N. (1978). Comparative evaluation of antiperoxidative action of flavonoids. Pharmacological Research Communications, 10, 133–136. 652840 10.1016/s0031-6989(78)80071-x

[fsn33217-bib-0028] Changa, L. W. , Yena, W. J. , Huangb, S. C. , & Duha, P. D. (2002). Antioxidant activity of sesame coat. Food Chemistry, 78, 347–354.

[fsn33217-bib-0030] Chen, C. C. , Kuo, M. C. , Wu, C. M. , & Ho, C. T. (1986). Pungent compounds of ginger (*Zingiber officinale* Roscoe) extracted by liquid carbon dioxide. Journal of Agricultural and Food Chemistry, 34(3), 477–480.

[fsn33217-bib-0031] Daglia, M. , Papetti, A. , Gregotti, C. , Berte, F. , & Gazzani, G. (2000). In vitro antioxidant and ex vivo protective activities of green and roasted coffee. Journal of Agricultural and Food Chemistry, 48, 1449–1454. 10820041 10.1021/jf990510g

[fsn33217-bib-0032] de la Rosa, L. A. , Alvarez‐Parrilla, E. , & Shahidi, F. (2011). Phenolic compounds and antioxidant activity of kernels and shells of Mexican pecan (*Carya illinoinensis*). Journal of Agricultural and Food Chemistry, 59(1), 152–162. 21138247 10.1021/jf1034306

[fsn33217-bib-0033] Del‐Castillo, M. D. , Ames, J. M. , & Gordon, M. H. (2002). Effect of roasting on the antioxidant activity of coffee brews. Journal of Agricultural and Food Chemistry, 50, 3698–3703. 12059145 10.1021/jf011702q

[fsn33217-bib-0034] do Prado, A. C. P. , Arragao, A. M. , Fett, R. , & Block, J. M. (2009). Antioxidant properties of Pecan nut [*Carya illinoinensis* (Wangenh.) C. Koch] Shell infusion. Grasas y Aceites, 60(4), 330–335.

[fsn33217-bib-0035] Docea, A. O. , Calina, D. , Buga, A. M. , Zlatian, O. , Paoliello, M. M. B. , Mogosanu, G. D. , Streba, C. T. , Popescu, E. L. , Stoica, A. E. , Birca, A. C. , Vasile, B. S. , Grumezescu, A. M. , & Mogoanta, L. (2020). The effect of silver nanoparticles on antioxidant/pro‐oxidant balance in a murine model. International Journal of Molecular Sciences, 21, 17. 10.3390/ijms21041233 PMC7072874 32059471

[fsn33217-bib-0036] Eberhardt, M. V. , Lee, C. , & Liu, R. (2000). Antioxidant activity of fresh apples. Nature, 405, 903–904. 10.1038/35016151 10879522

[fsn33217-bib-0037] Escribano, J. , Pedreno, M. A. , Garcia‐Carmona, F. , & Munoz, R. (1998). Characterization of the antiradical activity of betalains from Beta *Vulgaris L*. roots. Phytochemical Analysis, 9, 124–127.

[fsn33217-bib-0039] Farah, A. , & Donangelo, C. M. (2006). Phenolic compounds in coffee. Brazilian Journal of Plant Physiology, 18(1), 23–36.

[fsn33217-bib-0040] Franis, F. J. , & Anthocyanins, & Betalains . (1999). In F. J. Francis (Ed.), Colorants (pp. 55–66). Eagan Press.

[fsn33217-bib-0041] Fuhrman, B. , Rosenblat, M. , Hayek, T. , Coleman, R. , & Aviram, M. (2000). Ginger extract consumption reduces plasma cholesterol, inhibits LDL oxidation and attenuates development of atherosclerosis in atherosclerotic, apolipoprotein E‐deficient mice. Journal of Nutrition, 130, 1124–1131. 10801908 10.1093/jn/130.5.1124

[fsn33217-bib-0042] Fukumoto, L. R. , & Mazza, G. (2000). Assessing antioxidant and prooxidant activities of phenolic compounds. Journal of Agricultural and Food Chemistry, 48, 3597–3604. 10956156 10.1021/jf000220w

[fsn33217-bib-0043] Fuleki, T. , & Ricaardo‐da‐Silva, M. J. (2003). Effects of cultivar and processing method on the contents of catechins and procyanidins in grape juice. Journal of Agriculture and Food Chemistry, 51, 640–646. 10.1021/jf020689m 12537435

[fsn33217-bib-0044] Garcia‐Alonso, M. (2004). Evaluation of the antioxidante properties of fruits. Food Chemistry, 84, 13–18.

[fsn33217-bib-0045] Geil, P. B. , & Anderson, J. W. (1994). Nutrition and health implications of dry beans: A review. Journal of the American College of Nutrition, 13, 549–558. 7706585 10.1080/07315724.1994.10718446

[fsn33217-bib-0046] Ghisalberti, E. L. (1979). Propolis: A review. Bee World, 60, 59–84.

[fsn33217-bib-0047] Giovannucci, E. (1999). Tomatoes, tomato‐based products, lycopene, and cancer: Review of the epidemiological literature. Journal of the National Cancer Institute, 91, 317–331. 10050865 10.1093/jnci/91.4.317

[fsn33217-bib-0048] Gulcin, I. (2006). Antioxidant activity of caffeic acid (3, 4‐dihydroxycinnamic acid). Toxicology, 217, 213–220. 16243424 10.1016/j.tox.2005.09.011

[fsn33217-bib-0049] Gutteridge, J. M. C. , Rowley, D. A. , & Halliwell, B. (1981). Superoxide dependant formation of hydroxyl radicals in the presence of iron salts. The Biochemical Journal, 199, 263–265. 6175315 10.1042/bj1990263 PMC1163361

[fsn33217-bib-0050] Halliwell, B. (1993). DNA and free radicals (pp. 315–327). Horwood.

[fsn33217-bib-0052] Hannum, S. M. (2004). Potential impact of strawberries on human health: A review of the science. Critical Reviews in Food Science and Nutrition, 44, 1–17. 15077879 10.1080/10408690490263756

[fsn33217-bib-0053] Hanock, B. G. (1997). Texas Pecan handbook. Development of Pecan Industry, Texas Agricultural Extension Service.

[fsn33217-bib-0054] Heber, D. (2000). Colorful cancer prevention: A‐carotene, lycopene and lung cancer. American Journal of Clinical Nutrition, 72, 901–902. 11010928 10.1093/ajcn/72.4.901

[fsn33217-bib-0055] Heinrich, M. , Appendino, G. , Efferth, T. , Fürst, R. , Izzo, A. A. , Kayser, O. , Pezzuto, J. M. , & Viljoen, A. (2020). Best practice in research—Overcoming common challenges in phytopharmacological research. Journal of Ethnopharmacology, 246, 112230. 31526860 10.1016/j.jep.2019.112230

[fsn33217-bib-0056] Hertog, M. G. L. , Feskens, E. J. M. , Hollman, P. C. H. , Katan, M. B. , & Kromhout, D. (1993). Dietary antioxidant flavonoids and risk of coronary heart disease: The Zutphen Elderly Study. Lancet, 342, 1007–1011. 8105262 10.1016/0140-6736(93)92876-u

[fsn33217-bib-0057] Hirasa, K. , & Takemasa, M. (1998). Spice science and technology. Marcel Dekker.

[fsn33217-bib-0058] Hossain, R. , Quispe, C. , Herrera‐Bravo, J. , Islam, M. S. , Sarkar, C. , Islam, M. T. , Martorell, M. , Cruz‐Martins, N. , Al‐Harrasi, A. , Al‐Rawahi, A. , Sharifi‐Rad, J. , Ibrayeva, M. , Daştan, S. D. , Alshehri, M. M. , Calina, D. , & Cho, W. C. (2021). Lasia spinosa chemical composition and therapeutic potential: A literature‐based review. Oxidative Medicine and Cellular Longevity, 2021, 1602437. 34992714 10.1155/2021/1602437 PMC8727140

[fsn33217-bib-0059] Houghton, P. J. , Zarka, R. , de la Heras, B. , & Hoult, J. R. S. (1995). Fixed oil of *Nigella sativa* and derived thymoquinone inhibit eicosanoid generation in leukocytes and membrane lipid peroxidation. Planta Medica, 61, 33–36. 7700988 10.1055/s-2006-957994

[fsn33217-bib-0060] Hughes, J. S. , Ganthavorn, C. , & Wilson‐Sanders, S. (1997). Dry beans inhibit azoxymethane‐induced colon carcinogenesis in F344 rats. The Journal of Nutrition, 127, 2328–2333. 9405582 10.1093/jn/127.12.2328

[fsn33217-bib-0061] Islam, M. T. , Quispe, C. , El‐Kersh, D. M. , Shill, M. C. , Bhardwaj, K. , Bhardwaj, P. , Sharifi‐Rad, J. , Martorell, M. , Hossain, R. , Al‐Harrasi, A. , Al‐Rawahi, A. , Butnariu, M. , Rotariu, L. S. , Suleria, H. A. R. , Taheri, Y. , Docea, A. O. , Calina, D. , & Cho, W. C. (2021). A literature‐based update on *Benincasa hispida* (Thunb.) Cogn.: Traditional uses, nutraceutical, and phytopharmacological profiles. Oxidative Medicine and Cellular Longevity, 2021, 6349041. 34925698 10.1155/2021/6349041 PMC8683187

[fsn33217-bib-0062] Javanmardi, J. , Stushnoff, C. , Locke, E. , & Vivanco, J. M. (2003). Antioxidant activity and total phenolic content of Iranian Ocimum accessions. Food Chemistry, 83, 547–550.

[fsn33217-bib-0063] Jeng, J. H. , Chen, S. Y. , Liao, C. H. , Tung, Y. Y. , Lin, B. R. , Hahn, L. J. , & Chang, M. C. (2002). Modulation of platelet aggregation by areca nut and betle leaf ingredients: Roles of reactive oxygen species and cyclogenase. Free Radical Biology and Medicine, 32, 860–871. 11978487 10.1016/s0891-5849(02)00749-9

[fsn33217-bib-0064] Jhamandas, J. H. , Wie, M. B. , Harris, K. , Mactavish, D. , & Kar, S. (2005). Fucoidan inhibits cellular and neurotoxic effects of β‐amyloid (Aβ) in rat cholinergic basal forebrain neurons. The European Journal of Neuroscience, 21, 2649–2659. 15926913 10.1111/j.1460-9568.2005.04111.x

[fsn33217-bib-0065] Kanner, K. , Harel, S. , & Granit, R. (2001). Betalainss—A new class of dietary cationized antioxidants. Journal of Agricultural and Food Chemistry, 49, 5178–5185. 11714300 10.1021/jf010456f

[fsn33217-bib-0066] Kasote, D. M. , Katyare, S. S. , Hegde, M. V. , & Bae, H. (2015). Significance of antioxidant potential of plants and its relevance to therapeutic applications. International Journal of Biological Sciences, 11, 982–991. 26157352 10.7150/ijbs.12096 PMC4495415

[fsn33217-bib-0067] Kim, H. J. , Chang, S. C. , & Shim, Y. B. (2002). Cyclodextrin modified screen printed graphite electrodes for detection of phenols. Bulletin of the Korean Chemical Society, 23, 427–431.

[fsn33217-bib-0068] Knekt, P. , Jarvinen, R. , Reunanen, A. , & Maatela, J. (1996). Flavonoid intake and coronary mortality in Finland: A cohort study. British Medical Journal, 312, 478–481. 8597679 10.1136/bmj.312.7029.478 PMC2349921

[fsn33217-bib-0209] Konovalov, D. A. , Cáceres, E. A. , Shcherbakova, E. A. , Herrera‐Bravo, J. , Chandran, D. , Martorell, M. , Hasan, M. , Kumar, M. , Bakrim, S. , Bouyahya, A. , Cho, W. C. , Sharifi‐Rad, J. , Suleria, H. A. R. , & Calina, D. (2022). Eryngium caeruleum: an update on ethnobotany, phytochemistry and biomedical applications. Chinese Medicine, 17(1), 114. 10.1186/s13020-022-00672-x 36175969 PMC9523986

[fsn33217-bib-0069] Kornsteiner, M. , Wagner, K. H. , & Elmadfa, I. (2006). Tocopherols and total phenolics in 10 different nut types. Food Chemistry, 98, 381–387.

[fsn33217-bib-0070] Krasnovakii, A. A. , & Paramonava, L. I. (1983). Interaction of singlet oxygen with carotenoids: Rate constants of physical and chemical quenching. Biophysics, 28, 769–774.

[fsn33217-bib-0071] Kumazawa, S. , Hamasaka, T. , & Nakayama, T. (2004). Antioxidant activity of propolis of various geographic origins. Food Chemistry, 84, 329–339.

[fsn33217-bib-0072] Kumazawa, S. , Taniguchi, M. , Suzuki, Y. , Shimura, M. , Kwon, M. I. S. , & Nakayama, T. (2002). Antioxidant activity of polyphenols in carob pods. Journal of Agricultural and Food Chemistry, 50(2). 10.1021/jf010938r 11782210

[fsn33217-bib-0073] Laranjinha, J. , Vieira, O. , Madeira, V. , & Almeida, L. (1995). Two related phenolic antioxidants with opposite effects on vitamin E content in low density lipoproteins oxidized by ferrylmyoglobin: Consumption vs. regeneration. Archives of Biochemistry and Biophysics, 323, 373–381. 7487101 10.1006/abbi.1995.0057

[fsn33217-bib-0074] Li, L. , Tsao, R. , Yang, R. , Kramer, J. K. G. , & Hernandez, M. (2007). Fatty acid profiles, tocopheol contents, and antioxidant activities of heartnut (*Juglans ailanthiofolia* var. cordiformis) and Persian walnut (*Juglans regia* L.). Journal of Agricultural and Food Chemistry, 55, 1164–1169. 17253708 10.1021/jf062322d

[fsn33217-bib-0075] Li, L. , Tsao, R. , Yang, R. , Liu, C. M. , Zhu, H. H. , & Young, J. C. (2006). Polyphenolic profiles and antioxidant activities of heartnut (*Juglans ailanthifolia* var. cordiformis) and Persian walnut (*Juglans regia* L.). Journal of Agricultural and Food Chemistry, 54, 8033–8040. 17032006 10.1021/jf0612171

[fsn33217-bib-0077] Loliger, J. (1991). The use of antioxidants in food. In O. I. Aruoma & B. Halliwell (Eds.), Free radicals and food additives (pp. 129–150). Taylor and Francis.

[fsn33217-bib-0078] Mahmoud, A. , & Shaheen, R. (1996). Effects of the volatile oil of *Nigella sativa* seeds on the uterine smooth muscle of rat and Guinea pig. Journal of Ethnopharmacology, 52, 23–26. 8733115 10.1016/0378-8741(95)01330-x

[fsn33217-bib-0079] Mayne, S. T. (1996). Beta‐carotene, carotenoids, and disease prevention in humans. The FASEB Journal, 10, 690–701. 8635686

[fsn33217-bib-0080] Meir, S. , Kanner, J. , Akiri, B. , & Philosoph‐Hadas, S. (1995). Determination and involvement of aqueous reducing compounds in oxidative defense systems of various senescing leaves. Journal of Agricultural and Food Chemistry, 43, 1813–1819.

[fsn33217-bib-0081] Meyer, A. S. , Donovan, J. L. , Pearson, D. A. , Waterhouse, A. L. , & Frankel, E. N. (1998). Fruit hydroxycinnamic acids inhibit low density lipoprotein oxidation *in vitro* . Journal of Agricultural and Food Chemistry, 46, 1783–1787.

[fsn33217-bib-0082] Meyers, K. J. , Watkins, C. B. , Pritts, M. P. , & Liu, R. H. (2003). Antioxidant and antiproliferative activities of strawberries. Journal of Agricultural and Food Chemistry, 51, 6887–6892. 14582991 10.1021/jf034506n

[fsn33217-bib-0083] Mititelu, R. R. , Pădureanu, R. , Băcănoiu, M. , Pădureanu, V. , Docea, A. O. , Calina, D. , Barbulescu, A. L. , & Buga, A. M. (2020). Inflammatory and Oxidative Stress Markers‐Mirror Tools in Rheumatoid Arthritis. Biomedicine, 8(5), 125. 10.3390/biomedicines8050125 PMC7277871 32429264

[fsn33217-bib-0084] Munoz‐Espada, A. C. , Wood, K. V. , Bordelon, B. , & Watkins, B. A. (2004). Anthocyanin quantification and radical scavenging capacity of Concord, Norton, and Marechal Foch grapes and wines. Journal of Agricultural and Food Chemistry, 52(22), 6779–6786. 15506816 10.1021/jf040087y

[fsn33217-bib-0085] Nakatani, N. (1997). Antioxidants from spices and herbs. In F. Shahidi (Ed.), Natural antioxidants: Chemistry, health effects, and applications (pp. 64–75). AOCS Press.

[fsn33217-bib-0086] O'byrne, D. (2002). Comparison of the antioxidant effects of Concord grape juice flavonoids and R‐tocopherol on markers of oxidative stress in healthy adults. American Journal of Clinical Nutrition, 76, 1367–1374. 12450905 10.1093/ajcn/76.6.1367

[fsn33217-bib-0087] Oliveira, I. , Sousa, A. , Ferreira, I. C. F. R. , Bento, A. , Estevinho, L. , & Pereira, J. A. (2008). Total phenols, antioxidant potential and antimicrobial activity of walnut (*Juglans regia* L.) green husks. Food and Chemical Toxicology, 46, 2326–2331. 18448225 10.1016/j.fct.2008.03.017

[fsn33217-bib-0088] Olson, J. A. , & Krinsky, N. I. (1995). Introduction: The colorful fascinating world of the carotenoids: Important physiologic modulators. The FASEB Journal, 9, 1547–1550. 8529833 10.1096/fasebj.9.15.8529833

[fsn33217-bib-0089] Padureanu, R. , Albu, C. V. , Mititelu, R. R. , Bacanoiu, M. V. , Docea, A. O. , Calina, D. , Padureanu, V. , Olaru, G. , Sandu, R. E. , Malin, R. D. , & Buga, A.‐M. (2019). Oxidative stress and inflammation interdependence in multiple sclerosis. Journal of Clinical Medicine, 8, 1815. 31683787 10.3390/jcm8111815 PMC6912446

[fsn33217-bib-0090] Painuli, S. , Quispe, C. , Herrera‐Bravo, J. , Semwal, P. , Martorell, M. , Almarhoon, Z. M. , Seilkhan, A. , Ydyrys, A. , Rad, J. S. , Alshehri, M. M. , Daştan, S. D. , Taheri, Y. , Calina, D. , & Cho, W. C. (2022). Nutraceutical profiling, bioactive composition, and biological applications of *Lepidium sativum* L. Oxidative Medicine and Cellular Longevity, 2022, 2910411. 35096265 10.1155/2022/2910411 PMC8791756

[fsn33217-bib-0091] Patankar, M. S. , Oehninger, S. , Barnett, T. , Williams, R. L. , & Clark, G. F. (1993). A revised structure for fucoidan may explain some of its biological activities. The Journal of Biological Chemistry, 268, 21770–21776. 8408031

[fsn33217-bib-0092] Patra, J. K. , Das, G. , Fraceto, L. F. , Campos, E. V. R. , Rodriguez‐Torres, M. D. P. , Acosta‐Torres, L. S. , Diaz‐Torres, L. A. , Grillo, R. , Swamy, M. K. , Sharma, S. , Habtemariam, S. , & Shin, H.‐S. (2018). Nano based drug delivery systems: Recent developments and future prospects. Journal of Nanobiotechnology, 16, 71. 30231877 10.1186/s12951-018-0392-8 PMC6145203

[fsn33217-bib-0093] Pereira, J. A. , Oliveira, I. , Sousa, A. , Ferreira, I. C. F. R. , Bento, A. , & Estevinho, L. (2008). Bioactive properties and chemical composition of six walnut (*Juglans regia* L.) cultivars. Food and Chemical Toxicology, 46, 2103–2111. 18334279 10.1016/j.fct.2008.02.002

[fsn33217-bib-0094] Pereira, J. A. , Oliveira, I. , Sousa, A. , Valentao, P. , Andrade, P. B. , Ferreira, I. C. F. R. , Ferreres, F. , Bento, A. , Seabra, R. , & Estevinho, L. (2007). Walnut (*Juglans regia* L.) leaves: Phenolic compounds, antimicrobial activity and antioxidant potential of different cultivars. Food and Chemical Toxicology, 45, 2287–2295. 17637491 10.1016/j.fct.2007.06.004

[fsn33217-bib-0095] Ponce, N. M. A. , Pujol, C. A. , Damonte, E. B. , Flores, M. L. , & Stortz, C. A. (2003). Fucoidans from the brown seaweed Adenocystis utricularis: Extraction methods, antiviral activity and structural studies. Carbohydrate Research, 338, 153–165. 12526839 10.1016/s0008-6215(02)00403-2

[fsn33217-bib-0211] Popović‐Djordjević, J. , Quispe, C. , Giordo, R. , Kostić, A. , Katanić Stanković, J. S. , Tsouh Fokou, P. V. , Carbone, K. , Martorell, M. , Kumar, M. , Pintus, G. , Sharifi‐Rad, J. , Docea, A. O. , & Calina, D. (2022). Natural products and synthetic analogues against HIV: A perspective to develop new potential anti‐HIV drugs. European Journal of Medicinal Chemistry, 233, 114217. 10.1016/j.ejmech.2022.114217 35276425

[fsn33217-bib-0212] Quetglas‐Llabrés, M. M. , Quispe, C. , Herrera‐Bravo, J. , Catarino, M. D. , Pereira, O. R. , Cardoso, S. M. , Dua, K. , Chellappan, D. K. , Pabreja, K. , Satija, S. , Mehta, M. , Sureda, A. , Martorell, M. , Satmbekova, D. , Yeskaliyeva, B. , Sharifi‐Rad, J. , Rasool, N. , Butnariu, M. , Bagiu, I. C. , … Cho, W. C. (2022). Pharmacological Properties of Bergapten: Mechanistic and Therapeutic Aspects. Oxidative Medicine and Cellular Longevity, 2022, 8615242. 10.1155/2022/8615242 35509838 PMC9060977

[fsn33217-bib-0096] Quispe, C. , Herrera‐Bravo, J. , Javed, Z. , Khan, K. , Raza, S. , Gulsunoglu‐Konuskan, Z. , Daştan, S. D. , Sytar, O. , Martorell, M. , Sharifi‐Rad, J. , & Calina, D. (2022). Therapeutic applications of curcumin in diabetes: A review and perspective. BioMed Research International, 2022, 1375892. 35155670 10.1155/2022/1375892 PMC8828342

[fsn33217-bib-0100] Ramji, N. , Iyer, R. , & Chandrasekaran, S. (2002). Phenolic antibacterials from *Piper betle* in the prevention of halitosis. Journal of Ethnopharmacology, 83, 149–152. 12413722 10.1016/s0378-8741(02)00194-0

[fsn33217-bib-0101] Rao, A. V. , & Agarwal, S. (2000). Role of antioxidant lycopene in cancer and heart disease. Journal of American College of Nutrition, 19, 563–569. 10.1080/07315724.2000.10718953 11022869

[fsn33217-bib-0102] Riboli, E. , & Norat, T. (2003). Epidemiologic evidence of the protective effect of fruit and vegetables on cancer risk. The American Journal of Clinical Nutrition, 78, 559S–569S. 12936950 10.1093/ajcn/78.3.559S

[fsn33217-bib-0103] Rice‐Evans, C. A. , Miller, N. J. , Bolwell, P. G. , Bramley, P. M. , & Pridham, J. B. (1995). Antioxidant potential of intermediates in phenylpropanoid metabolism in higher plants. Free Radical Research, 22, 375–383. 7633567 10.3109/10715769509145649

[fsn33217-bib-0105] Ruby, A. J. , Kuttan, G. , Babu, K. D. , Rajasekharan, K. N. , & Kutta, R. (1995). Anti‐tumour and antioxidant activity of natural curcuminoids. Cancer Letters, 94(1), 79–83. 7621448 10.1016/0304-3835(95)03827-j

[fsn33217-bib-0106] Salehi, B. , Calina, D. , Docea, A. O. , Koirala, N. , Aryal, S. , Lombardo, D. , Pasqua, L. , Taheri, Y. , Castillo, C. M. S. , Martorell, M. , Martins, N. , Iriti, M. , Suleria, H. A. R. , & Sharifi‐Rad, J. (2020). Curcumin's nanomedicine formulations for therapeutic application in neurological diseases. Journal of Clinical Medicine, 9, 35. 10.3390/jcm9020430 PMC7074182 32033365

[fsn33217-bib-0107] Salehi, B. , Jornet, P. L. , Lopez, E. P. F. , Calina, D. , Sharifi‐Rad, M. , Ramirez‐Alarcon, K. , Forman, K. , Fernandez, M. , Martorell, M. , Setzer, W. N. , Martins, N. , Rodrigues, C. F. , & Sharifi‐Rad, J. (2019). Plant‐derived bioactives in oral mucosal lesions: A key emphasis to curcumin, lycopene, chamomile, aloe vera, green tea and coffee properties. Biomolecules, 9, 23. 30884918 10.3390/biom9030106 PMC6468600

[fsn33217-bib-0108] Salehi, B. , Prakash Mishra, A. , Nigam, M. , Karazhan, N. , Shukla, I. , Kiełtyka‐Dadasiewicz, A. , Sawicka, B. , Głowacka, A. , Abu‐Darwish, M. S. , Hussein Tarawneh, A. , Gadetskaya, A. V. , Cabral, C. , Salgueiro, L. , Victoriano, M. , Martorell, M. , Docea, A. O. , Abdolshahi, A. , Calina, D. , & Sharifi‐Rad, J. (2021). Ficus plants: State of the art from a phytochemical, pharmacological, and toxicological perspective. Phytotherapy Research, 35, 1187–1217. 33025667 10.1002/ptr.6884

[fsn33217-bib-0109] Sandberg, A. S. (2000). Developing functional ingredients a case study. In Functional foods: Concept to product. Elsevier.

[fsn33217-bib-0110] Saravanan, R. , Prakasam, A. , Ramesh, B. , & PUGAlendi, K. V. (2002). Influence of *Piper betle* on hepatic marker enzymes and tissue antioxidant status in ethanol‐treated Wister rats. Journal of Medicinal Food, 5, 197–204. 12639394 10.1089/109662002763003348

[fsn33217-bib-0213] Scheau, C. , Badarau, I. A. , Mihai, L. G. , Scheau, A. E. , Costache, D. O. , Constantin, C. , Calina, D. , Caruntu, C. , Costache, R. S. , & Caruntu, A. (2020). Cannabinoids in the Pathophysiology of Skin Inflammation. Molecules, 25(3), 652. 10.3390/molecules25030652 32033005 PMC7037408

[fsn33217-bib-0111] Shahidi, F. , Amarowicz, R. , Abou‐Gharbia, H. A. , & Shehata, A. Y. (1997). Endgenous antioxidants and stability of sesame oil as affected by processing and storage. Journal of the American Oil Chemists' Society, 74, 147–148.

[fsn33217-bib-0118] Sharifi‐Rad, M. , Kumar, N. V. A. , Zucca, P. , Varoni, E. M. , Dini, L. , Panzarini, E. , Rajkovic, J. , Fokou, P. V. T. , Azzini, E. , Peluso, I. , Mishra, A. P. , Nigam, M. , El Rayess, Y. , EL Beyrouthy, M. , Polito, L. , Iriti, M. , Martins, N. , Martorell, M. , Docea, A. O. , … Sharifi‐Rad, J. (2020). Lifestyle, oxidative stress, and antioxidants: Back and forth in the pathophysiology of chronic diseases. Frontiers in Physiology, 11, 21. 32714204 10.3389/fphys.2020.00694 PMC7347016

[fsn33217-bib-0117] Sharifi‐Rad, J. , Rodrigues, C. F. , Sharopov, F. , Docea, A. O. , Karaca, A. C. , Sharifi‐Rad, M. , Kahveci Karincaoglu, D. , Gulseren, G. , Senol, E. , Demircan, E. , Taheri, Y. , Suleria, H. A. R. , Ozcelik, B. , Kasapoglu, K. N. , Gultekin‐Ozguven, M. , Daskaya‐Dikmen, C. , Cho, W. C. , Martins, N. , & Calina, D. (2020). Diet, lifestyle and cardiovascular diseases: Linking pathophysiology to cardioprotective effects of natural bioactive compounds. International Journal of Environmental Research and Public Health, 17, 31. 10.3390/ijerph17072326 PMC7177934 32235611

[fsn33217-bib-0112] Sharifi‐Rad, J. , Dey, A. , Koirala, N. , Shaheen, S. , El Omari, N. , Salehi, B. , Goloshvili, T. , Cirone Silva, N. C. , Bouyahya, A. , Vitalini, S. , Varoni, E. M. , Martorell, M. , Abdolshahi, A. , Docea, A. O. , Iriti, M. , Calina, D. , Les, F. , López, V. , & Caruntu, C. (2021). Cinnamomum species: Bridging phytochemistry knowledge, pharmacological properties and toxicological safety for health benefits. Frontiers in Pharmacology, 12, 600139. 34045956 10.3389/fphar.2021.600139 PMC8144503

[fsn33217-bib-0115] Sharifi‐Rad, J. , Quispe, C. , Herrera‐Bravo, J. , Akram, M. , Abbaass, W. , Semwal, P. , Painuli, S. , Konovalov, D. A. , Alfred, M. A. , Kumar, N. V. A. , Imran, M. , Nadeem, M. , Sawicka, B. , Pszczółkowski, P. , Bienia, B. , Barbaś, P. , Mahmud, S. , Durazzo, A. , Lucarini, M. , … Calina, D. (2021). Phytochemical constituents, biological activities, and health‐promoting effects of the Melissa officinalis. Oxidative Medicine and Cellular Longevity, 2021, 6584693. 10.1155/2021/6584693 PMC11283336 39071243

[fsn33217-bib-0116] Sharifi‐Rad, J. , Quispe, C. , Imran, M. , Rauf, A. , Nadeem, M. , Gondal, T. A. , Ahmad, B. , Atif, M. , Mubarak, M. S. , Sytar, O. , Zhilina, O. M. , Garsiya, E. R. , Smeriglio, A. , Trombetta, D. , Pons, D. G. , Martorell, M. , Cardoso, S. M. , Razis, A. F. A. , Sunusi, U. , … Calina, D. (2021). Genistein: An integrative overview of its mode of action, pharmacological properties, and health benefits. Oxidative Medicine and Cellular Longevity, 2021, 3268136. 34336089 10.1155/2021/3268136 PMC8315847

[fsn33217-bib-0113] Sharifi‐Rad, J. , Quispe, C. , Bouyahya, A. , El Menyiy, N. , El Omari, N. , Shahinozzaman, M. , Ara Haque Ovey, M. , Koirala, N. , Panthi, M. , Ertani, A. , Nicola, S. , Lapava, N. , Herrera‐Bravo, J. , Salazar, L. A. , Changan, S. , Kumar, M. , & Calina, D. (2022). Ethnobotany, phytochemistry, biological activities, and health‐promoting effects of the genus Bulbophyllum. Evidence‐based Complementary and Alternative Medicine, 2022, 6727609. 35295925 10.1155/2022/6727609 PMC8920616

[fsn33217-bib-0114] Sharifi‐Rad, J. , Quispe, C. , Durazzo, A. , Lucarini, M. , Souto, E. B. , Santini, A. , Imran, M. , Moussa, A. Y. , Mostafa, N. M. , El‐Shazly, M. , Sener, B. , Schoebitz, M. , Martorell, M. , Dey, A. , Calina, D. , & Cruz‐Martins, N. (2022). Resveratrol' biotechnological applications: Enlightening its antimicrobial and antioxidant properties. Journal of Herbal Medicine, 32, 100550.

[fsn33217-bib-0216] Sharifi‐Rad, J. , Quispe, C. , Turgumbayeva, A. , Mertdinç, Z. , Tütüncü, S. , Aydar, E. F. , Özçelik, B. , Anna, S. W. , Mariola, S. , Koziróg, A. , Otlewska, A. , Antolak, H. , Sen, S. , Acharya, K. , Lapava, N. , Emamzadeh‐Yazdi, S. , Martorell, M. , Kumar, M. , Varoni, E. M. , … Calina, D. (2022a). *Santalum* Genus: phytochemical constituents, biological activities and health promoting‐effects. Zeitschrift fuer Naturforschung, C: Journal of Biosciences. 10.1515/znc-2022-0076 36069757

[fsn33217-bib-0214] Sharifi‐Rad, J. , Herrera‐Bravo, J. , Kamiloglu, S. , Petroni, K. , Mishra, A. P. , Monserrat‐Mesquida, M. , Sureda, A. , Martorell, M. , Aidarbekovna, D. S. , Yessimsiitova, Z. , Ydyrys, A. , Hano, C. , Calina, D. , & Cho, W. C. (2022b). Recent advances in the therapeutic potential of emodin for human health. Biomedicine & Pharmacotherapy, 154, 113555. 10.1016/j.biopha.2022.113555 36027610

[fsn33217-bib-0215] Sharifi‐Rad, J. , Quispe, C. , Kumar, M. , Akram, M. , Amin, M. , Iqbal, M. , Koirala, N. , Sytar, O. , Kregiel, D. , Nicola, S. , Ertani, A. , Victoriano, M. , Khosravi‐Dehaghi, N. , Martorell, M. , Alshehri, M. M. , Butnariu, M. , Pentea, M. , Rotariu, L. S. , Calina, D. , … Cho, W. C. (2022c). Hyssopus Essential Oil: An Update of Its Phytochemistry, Biological Activities, and Safety Profile. Oxidative Medicine and Cellular Longevity, 2022, 8442734. 10.1155/2022/8442734 35069979 PMC8776447

[fsn33217-bib-0119] Sharma, O. P. (1976). Antioxidant activity of curcumin and related compounds. Biochemical Pharmacology, 25, 1811–1812. 942483 10.1016/0006-2952(76)90421-4

[fsn33217-bib-0218] Sharma, E. , Attri, D. C. , Sati, P. , Dhyani, P. , Szopa, A. , Sharifi‐Rad, J. , Hano, C. , Calina, D. , & Cho, W. C. (2022). Recent updates on anticancer mechanisms of polyphenols. Frontiers in Cell and Development Biology, 10, 1005910. 10.3389/fcell.2022.1005910 PMC9557130 36247004

[fsn33217-bib-0120] Simon, J. E. , Mrales, M. R. , Phippen, W. B. , Vieira, R. F. , & Hao, Z. (1999). Basil: A source of aroma compounds and a popular culinary and ornamental herb. In J. Janick (Ed.), Perspectives on new crops and new uses (pp. 499–505). ASHS Press.

[fsn33217-bib-0121] Singleton, V. L. , & Rossi, J. A. (1965). Colorimetry of total phenolics with phosphomolybdic‐phosphotungstic acid reagents. American Journal of Enology and Viticulture, 16(3), 144–158.

[fsn33217-bib-0123] Stahl, W. , & Sies, H. (2003). Antioxidant activity of carotenoids. Molecular Aspects of Medicine, 24, 345–335. 14585305 10.1016/s0098-2997(03)00030-x

[fsn33217-bib-0124] Stampar, F. , Solar, A. , Hudina, M. , Veberic, R. , & Colaric, M. (2006). Traditional walnut liqueur—Cocktail of phenolics. Food Chemistry, 95, 627–631.

[fsn33217-bib-0125] Steinhart, H. , Luger, A. , & Piost, J. (2001). *Proceedings of 19th International Scientific Colloquium on Coffee*, Trieste, 14.‐18.5.

[fsn33217-bib-0126] Stoilova, I. , Krastanov, A. , Stoyanova, A. , Denev, P. , & Gargova, S. (2007). Antioxidant activity of a ginger extract (*Zingiber officinale*). Food Chemistry, 102(3), 764–770.

[fsn33217-bib-0127] Stwart, A. J. , Bozonnet, S. , Mullen, W. , Jenkines, G. I. , Lean, M. E. J. , & Crozier, A. (2000). Occurrence of flavonols in tomatoes and tomato‐based products. Journal of Agricultural and Food Chemistry, 48, 2663–2669. 10898604 10.1021/jf000070p

[fsn33217-bib-0128] Taheri, Y. , Quispe, C. , Herrera‐Bravo, J. , Sharifi‐Rad, J. , Ezzat, S. M. , Merghany, R. M. , Shaheen, S. , Azmi, L. , Prakash Mishra, A. , Sener, B. , KıLıÇ, M. , Sen, S. , Acharya, K. , Nasiri, A. , Cruz‐Martins, N. , Tsouh Fokou, P. V. , Ydyrys, A. , Bassygarayev, Z. , Daştan, S. D. , … Cho, W. C. (2022). Urtica dioica‐derived phytochemicals for pharmacological and therapeutic applications. Evidence‐based Complementary and Alternative Medicine, 2022, 4024331. 35251206 10.1155/2022/4024331 PMC8894011

[fsn33217-bib-0129] Tehila, T. S. , Margalit, B. , Dorit, V. M. , Shlomo, G. , & Shoshana, A. (2005). Antioxidant activity of the polysaccharide of the red microalga *Porphyridium* sp. Journal of Applied Phycology, 17, 215–222.

[fsn33217-bib-0130] Teissedre, P. L. , Frankel, E. N. , Waterhouse, A. L. , Peleg, H. , & German, J. B. (1996). Inhibition of in‐vitro human LDL oxidation by phenolic antioxidants from grapes and wines. Journal of the Science of Food and Agriculture, 70, 55–61.

[fsn33217-bib-0132] The PlantList . http://www.theplantlist.org/

[fsn33217-bib-0133] The Wealth of India . (1969). A dictionary of Indian raw materials and industrial products (Vol. VIII). Publications and Information Directorate, CSIR.

[fsn33217-bib-0134] Thompson, L. U. (1994). Antioxidants and hormone‐mediated health benefits of whole grains. Critical Reviews in Food Science & Nutrition, 34(5–6), 473–497. 7811379 10.1080/10408399409527676

[fsn33217-bib-0135] Toor, R. K. , & Savage, G. P. (1992). Antioxidant activity in different fractions of tomatoes. Food Research International, 38(5), 487–494.

[fsn33217-bib-0219] Tsoukalas, D. , Fragkiadaki, P. , Docea, A. O. , Alegakis, A. K. , Sarandi, E. , Vakonaki, E. , Salataj, E. , Kouvidi, E. , Nikitovic, D. , Kovatsi, L. , Spandidos, D. A. , Tsatsakis, A. , & Calina, D. (2019a). Association of nutraceutical supplements with longer telomere length. International Journal of Molecular Medicine, 44(1), 218–226. 10.3892/ijmm.2019.4191 31115552 PMC6559326

[fsn33217-bib-0220] Tsoukalas, D. , Fragoulakis, V. , Sarandi, E. , Docea, A. O. , Papakonstaninou, E. , Tsilimidos, G. , Anamaterou, C. , Fragkiadaki, P. , Aschner, M. , Tsatsakis, A. , Drakoulis, N. , & Calina, D. (2019b). Targeted Metabolomic Analysis of Serum Fatty Acids for the Prediction of Autoimmune Diseases. Frontiers in Molecular Biosciences, 6, 120. 10.3389/fmolb.2019.00120 31737644 PMC6839420

[fsn33217-bib-0221] Tsoukalas, D. , Zlatian, O. , Mitroi, M. , Renieri, E. , Tsatsakis, A. , Izotov, B. N. , Burada, F. , Sosoi, S. , Burada, E. , Buga, A. M. , Rogoveanu, I. , Docea, A. O. , & Calina, D. (2021). A Novel Nutraceutical Formulation Can Improve Motor Activity and Decrease the Stress Level in a Murine Model of Middle‐Age Animals. Journal of Clinical Medicine, 10(4), 624. 10.3390/jcm10040624 33562115 PMC7915416

[fsn33217-bib-0136] Valko, M. , Izakovic, M. , Mazur, M. , Rhodes, C. J. , & Telser, J. (2004). Role of oxygen radicals in DNA damage and cancer incidence. Molecular and Cellular Biochemistry, 266, 37–56. 15646026 10.1023/b:mcbi.0000049134.69131.89

[fsn33217-bib-0222] Varela, C. , Melim, C. , Neves, B. G. , Sharifi‐Rad, J. , Calina, D. , Mamurova, A. , & Cabral, C. (2022). Cucurbitacins as potential anticancer agents: new insights on molecular mechanisms. Journal of Translational Medicine, 20(1), 630. 10.1186/s12967-022-03828-3 36585670 PMC9805216

[fsn33217-bib-0138] Wang, J. , Zhang, Q. , Zhang, Z. , & Li, Z. (2008). Antioxidant activity of sulfated polysaccharide fractions extracted from *Laminaria japonica* . International Journal of Biological Macromolecules, 42, 127–132. 18023861 10.1016/j.ijbiomac.2007.10.003

[fsn33217-bib-0139] Wangensteen, H. , Samuelsen, A. B. , & Malterud, K. E. (2004). Antioxidant activity in extracts from coriander. Food Chemistry, 88, 293–297. 487–494.

[fsn33217-bib-0223] Waterhouse, A. L. , Shirley, J. R. , Donovan, J. L . (1996). Antioxidants in chocolate. Lancet. 348(9030), 834. doi: 10.1016/S0140-6736(05)65262-2. PMID: 8814019. 8814019

[fsn33217-bib-0140] Wichtl, M. W. (1994). Herbal drugs and phytopharmaceuticals. Medpharm GmbH Scientific Publishers.

[fsn33217-bib-0141] Yousif, A. K. , & Alghzawi, H. M. (2000). Processing and characterization of carob powder. Food Chemistry, 69, 283–287.

[fsn33217-bib-0143] Zhuang, C. , Itoh, H. , Mizuno, T. , & Ito, H. (1995). Antitumor active fucoidan from the brown seaweed, umitoranoo (*Sargassum thunbergii*). Bioscience, Biotechnology, and Biochemistry, 59, 563–567. 7772818 10.1271/bbb.59.563

